# Overemphasizing individual differences and overlooking systemic factors reinforces educational inequality

**DOI:** 10.1038/s41539-023-00164-z

**Published:** 2023-05-08

**Authors:** Allison Zengilowski, Irum Maqbool, Surya Pratap Deka, Jesse C. Niebaum, Diego Placido, Benjamin Katz, Priti Shah, Yuko Munakata

**Affiliations:** 1 University of California, Davis, CA USA; 2 University of Cambridge, Cambridge, UK; 3 Virginia Tech, Blacksburg, VI USA; 4 University of Michigan, Ann Arbor, MI USA

**Keywords:** Education, Psychology

## Introduction

Imagine a student required to spend 30 minutes engaging in computerized training to improve their working memory capacity and ability to stay focused. They may practice tasks that are decontextualized from their classroom practice: store and recall an increasing amount of numbers, sequences of objects, and positions of different symbols^1^. After finishing computer training, the student’s class takes part in a well-being practice as part of their social-emotional learning (SEL) curriculum. The class is given instruction on mindful breathing to control temper during conflicts and showing kindness to others to improve intrinsic motivation, social-emotional competencies, and academic performance^2–4^. Students are told these activities will help them to succeed in their classes and everyday life. At the end of the day, the student walks past school police officers who are paid similarly to their classroom teachers, finds standardized test results in the mail that place them below average, and opens an empty fridge.

When considering inequalities in education, researchers are making earnest attempts at advancing student achievement and well-being. However, many existing or proposed interventions fail to account for a learner’s contextual realities, including structural and systemic barriers such as poverty and marginalization^5,6^, resulting in (re)producing a deficit discourse^7,8^. Individual trainings alone (which meta-analyses indicate do not translate well to academic domains^9–13^; see mixed results for SEL^3,14^) are unlikely to overcome the impact of broader inequities in and out of the classroom on student outcomes. Moreover, interventions established in the Euro-American context are being applied globally in contexts with unique systemic barriers to academic success. In doing so, there is little grounding in the needs of schools and students that, in theory, would benefit from these interventions the most. Continued emphasis on acontextual interventions serves to displace focus on the social responsibility that upholds systemic inequities.

When discussing structural and systemic barriers, we are referring to the ways that institutions, policies, and conditions have been created that reify oppression, domination, discrimination, and inequities^15^. For example, while some may see racial achievement gaps in the U.S. as a failing of individuals and schools to be rectified through “hard work,” many scholars and educators identify how the U.S. education *system* is “creating gaps between racial groups as well as disparate opportunities in education and employment.”^16^ And in India and Pakistan, individual approaches such as remedial learning classes are not seen as effective ways to address low academic attainment resulting from the intersectionality of socioeconomic and gender disparities. Rather, there are calls for improving government schools so those most at risk of low learning levels can benefit from structural improvements^17^. We argue that psychological scientists’ focus on interventions that target individual differences results in an underappreciation of structural factors and shapes the perceptions of what causes inequities in education in favor of focusing on the individual^18^. As a result, we consider the importance of context in education, critique the “universality” of interventions, and argue for approaches that prioritize structural focuses and local knowledge.

## Perspectives

### Contextual

Horace Mann described education as the “great equalizer” for social inequity^19^. While education may indeed offer this potential, we have seen in the 175 years since this statement how public education has also reinforced inequities^20^. In the U.S., the legacies of historical foundations perpetuate current inequitable education systems. Institutional resources determine student achievement. District funding disfavors expenditures for Black and Latine students^21^. When institutional resources are directed toward school policing and zero-tolerance policies, expulsion/suspension rates increase and test scores decrease for low-income Black, Hispanic, and Latine students, who then graduate and enroll in college at lower rates^22–24^. Racial incongruence between students and teachers due to a predominantly White teaching population in the U.S.^25^ is linked with lower achievement scores^26^, teacher bias against students of color^27^, lower teacher expectations^28^, differential tracking^29^, and underrepresentation of students of color in gifted programs^30^. Curricula and assessments are not designed for minoritized students. Standard measures of achievement overlook the hidden assets of disenfranchised youth (e.g., creativity and non-essentialist thinking as strengths of low-socioeconomic status (SES) students, despite researchers often identifying such students as having poor executive function; low-SES students show better empathy, attentiveness to others, ability to work in groups, and, in some cases, better executive functions under stress^31–33^). Content and pedagogical approaches often are disparate from the lived experiences of Black and Latine students, which is problematic given the importance of relevance for achievement motivation^34^.

In the Global South, structural and social inequality are persistent issues. Students face differential access to formal schooling, wide ranges of teaching quality, inequitable school resources, gender discrimination, and linguistic barriers^5,6^. In addition, they may be privy to widespread poverty and intersectional disadvantages across gender, disability, caste, ethnic, and regional axes^17,35,36^. COVID-19 and climate risks have worsened these inequalities, such that marginalized children are less likely to remain in the educational system^37^.

### Empirical

While some research elevates the importance of self-control^38^ and genetic factors^39,40^ in predicting educational outcomes, we suggest that individual-difference studies also highlight the influence of environmental factors. Environmental factors such as social support influence performance on early childhood measures of self-control, show stability across the lifespan, and modulate the relationship between childhood self-control measures and life outcomes. For example, longitudinal links between the preschool delay of gratification and adolescent academic outcomes disappear after accounting for social support^41^. Estimates of heritability vary based on environmental conditions^40^. Societies with greater barriers to educational attainment show lower heritability estimates for educational attainment because structural constraints play a larger role in determining outcomes^42,43^. Polygenic scores (e.g., that predict educational attainment) are typically derived from White samples and address variations within those samples; they do not speak to variations between groups and typically fail to generalize to diverse groups by race, age, gender, or SES^44,45^. Individual-difference studies thus reliably highlight the importance of context and the problems with ascribing variations among people to personal factors considered in isolation.

### Circulating empiricism

Even with an understanding of the importance of context, psychological knowledge and evidence circulate through education systems around the world as “universally applicable,”^46^ with interventions exported to contexts outside of those in which they were developed^47^. An area where this is prominent is in social-emotional learning (SEL). SEL interventions often focus on learning skills for managing emotions, goal-setting, empathy, positive relationships, and decision-making^48,49^, which are considered important for achievement and lifelong learning.

Under the SEL umbrella is the practice of well-being, seen as a route to tackle educational inequality^50^ through supporting children in learning and enhancing skills necessary for coping and succeeding in a world in flux^51^. The designing and implementation of well-being curricula is in vogue in many contexts around the world. School-based well-being initiatives tend to draw on individualistic, universalized, and context-free notions^50,52^. Scaling programs based on these premises can promote a dangerous deficit discourse for disadvantaged schools and communities^50,53^, commit epistemic injustice^54,55^, and hinder creativity in addressing social-emotional needs of the community^56^.

Well-being curricula in South Asia^57,58^, assumes that well-being is a quality of the individual that can be enhanced with training and practice^59^. The effectiveness of these initiatives, predominantly conceptualized and tested in Euro-American contexts, reflects a Western-centric understanding of normative human development;^52,60^ The conceptualization of well-being itself is often confusing, with a variety of different disciplinary influences affecting the implementation and focus of programs aimed at well-being^50^. Imposing a Western-centric understanding of normative human development and well-being on people in different cultural contexts undermines local conceptualizations and practices related to well-being, (re)creating hierarchies of knowledge. Such practices, whether implemented in South Asia or with marginalized communities in the U.S.^61^, can overlook the collective affordances offered by the school as a relational place and hinder the creation of spaces that could have a meaningful impact on students’ well-being. Although these well-being initiatives promise to accrue benefits, they can shift focus away from structural inequality in education to individual capabilities instead^62^. Such a shift could pathologize marginalized and disadvantaged communities.

### Beyond the allure of individual solutions

Many panaceas have been proposed to reduce educational inequalities. We argue that engaging students in short cognitive, social–emotional, and other well-being trainings will not overcome formative experiences shaped by a lack of resources and opportunities at their schools. Will practicing well-being training, like mindfulness, during the school day really support students coping with traumatic stresses (or more than dedicated school counselors)? Individual interventions are easier for testing theories and constructs than implementing large structural changes; however, even large-scale executive function training programs have not benefited classroom skills or social-emotional outcomes without incorporating additional relevant support for targeted outcomes^63^. Large-scale randomized controlled trials have shown that mindfulness trainings do not benefit overall well-being and social-emotional functioning^4^ and can harm those most at risk for mental health issues^64^. Individual trainings are logical steps for researchers to explore and are palatable for policymakers but burdens already under-resourced teachers and displaces responsibility for the larger systemic changes required to reduce educational inequalities.

## A measured approach

It would be unfair to expect individual training to solve issues such as inadequate resources and opportunity ceilings, just as it would be unfair to expect structural changes alone to support students in staying focused, regulating emotions, and problem-solving. There are promising findings and ideas from individual approaches to executive function development and well-being, including steps to be sensitive to local contexts and support practice opportunities in ways that are relevant to those communities^65–68^. However, vast amounts of time and resources are extended to studies of individual solutions. For example, over 14,682 empirical articles on mindfulness were published between 1966 and 2021, with an exponential increase in recent years, leading to roughly 2400 empirical articles published in 2020 alone^69^. This overwhelming effort in testing mindfulness interventions has served to overshadow the structural factors that play a strong role in determining outcomes, such as SES. In the U.S., variance in student achievement is explained more by family economic background than school-level expenditures, suggesting that reducing inequality in life conditions may be necessary to secure better academic outcomes^70,71^. Higher overall school SES and positive school climate are associated with better overall student outcomes^72,73^, even though family SES remains a strong predictor of individual differences. At a more granular level, those higher in SES often exist in spaces fostering greater personal agency, which may lead them to conceive of inequality as related to individual traits or genetic factors. This frame moves higher SES individuals to de-emphasize structural and systemic issues tied to inequalities^74,75^. In the U.S., higher SES families may reinforce structural advantages by enrolling in private schools, eroding community and financial support for public education^76^. Focusing on individual differences can be important for developing science and policy; however, we argue for the necessity of considering the structural and systemic factors underlying or influencing the development of these differences.

To best serve the needs of local communities and those with whom we conduct research, a structural focus or combined approach may be most impactful. Though the list is not exhaustive, we refer to contextually sensitive programs that have supported individual needs by addressing structural issues while ensuring community participation. We acknowledge the debates surrounding the effectiveness or continuity of some of these programs and do not argue for generalizability or universality. The Child-Parent Center Program was initially started by a local superintendent in Chicago who emphasized developing culturally relevant instructional approaches, fostering parental involvement, and creating other structural changes, including smaller class sizes and increased attention to health/nutrition^77^. These changes led to higher high school and postsecondary completion^78,79^, increased earning capacity^80^, and lower rates of special education placements^81^, grade retention^82^, child maltreatment^83^, and juvenile and violent crime arrests^81,84^. Restorative Justice for Oakland Youth worked with their local school district to transform a focus on punitive policies toward restorative practices, leading to reduced suspensions and violence, an interruption in the school-to-prison pipeline, and increased youth agency^85^. In Delhi, an intervention engaging lay counselors from the local community showed sustained effectiveness in supporting the mental health needs of students in low-resourced settings^86^. And in Pakistan, a program focused on engaging youth in delivering early childhood education curricula increased the school readiness of rural children^87^. The Mid-Day Meal Scheme (MDMS), a program sponsored by the Indian government in Rajasthan, brings children across caste, class, gender, and religion divides to “sit together and share a common meal.” MDMS has improved enrollment, attendance, and nutritional status of girls and children from oppressed castes and tribes^88^. Moreover, MDMS strengthens school-community links and energizes local economies by engaging community members in the program^89^. A similar school lunch program implemented in 29 of the poorest rural districts in Pakistan increased girls’ school enrollment by 40% and engaged women in local communities, who purchased food and prepared meals^90,91^.

## Discussion

In the interventional and individual-difference research being conducted, we see merits to the ideas but harms in their implementations. Schools and classrooms should not be viewed solely as sites for testing; rather, they should be foundational to the work we conduct. Research–practice partnerships, which prioritize local knowledge, may offer a blueprint for this approach^92,93^. Creating meaningful and lasting relationships with districts and teachers can result in the production of responsible research and sustainable change at the structural level, whereas an individualistic focus could lead to internalization of blame for students facing structural inequality and disadvantages. Educational experiences are multidimensional, and an emphasis on individualistic, universal, and context-free notions of well-being and executive function fails to take into account the complexities of educational processes and structures. South Asia and the United States have multiple axes along which stratification and structural inequity exist and operate. Nevertheless, there is a rich history of cultural understandings of well-being and learning that can contribute to our collective understanding of student and community development^94^. We argue that communities are reservoirs of knowledge that can be used for supporting human flourishing. Therefore, the concepts we explore, such as executive function and well-being, should be operationalized in ways that reflect the lived realities of communities, and this conceptualization should account for structural issues that impede individual achievement and skill enhancement. It is imperative to balance our focus on individual skills alongside broader concerns related to structure to reshape our scientific thinking and real-world applications to create sustainable outcomes.
